# A Novel Fault-Tolerant Navigation and Positioning Method with Stereo-Camera/Micro Electro Mechanical Systems Inertial Measurement Unit (MEMS-IMU) in Hostile Environment

**DOI:** 10.3390/mi9120626

**Published:** 2018-11-27

**Authors:** Cheng Yuan, Jizhou Lai, Pin Lyu, Peng Shi, Wei Zhao, Kai Huang

**Affiliations:** 1 Navigation Research Center, College of Automation Engineering, Nanjing University of Aeronautics and Astronautics, Nanjing 211100, China; ycauto@nuaa.edu.cn (C.Y.); lvpin@nuaa.edu.cn (P.L.); ship@nuaa.edu.cn (P.S.); zhwac@nuaa.edu.cn (W.Z.); 2 Key Laboratory of Internet of Things and Control Technology in Jiangsu province, Nanjing University of Aeronautics and Astronautics, Nanjing 211100, China; 3 Shanxi Baocheng Aviation Instrument Co., Ltd., AVIC, Baoji 721006, China; bj212hk@163.com

**Keywords:** stereo visual-inertial odometry, fault tolerant, hostile environment, MEMS-IMU

## Abstract

Visual odometry (VO) is a new navigation and positioning method that estimates the ego-motion of vehicles from images. However, VO with unsatisfactory performance can fail severely in hostile environment because of the less feature, fast angular motions, or illumination change. Thus, enhancing the robustness of VO in hostile environment has become a popular research topic. In this paper, a novel fault-tolerant visual-inertial odometry (VIO) navigation and positioning method framework is presented. The micro electro mechanical systems inertial measurement unit (MEMS-IMU) is used to aid the stereo-camera, for a robust pose estimation in hostile environment. In the algorithm, the MEMS-IMU pre-integration is deployed to improve the motion estimation accuracy and robustness in the cases of similar or few feature points. Besides, a dramatic change detector and an adaptive observation noise factor are introduced, tolerating and decreasing the estimation error that is caused by large angular motion or wrong matching. Experiments in hostile environment showing that the presented method can achieve better position estimation when compared with the traditional VO and VIO method.

## 1. Introduction

Visual navigation is an emerging technology that uses camera to capture images of the surrounding environment and processes these images to estimate ego-motion, recognize path, and make navigation decisions. The visual sensor is mature, low-cost and widely-used in robotics. Given that visual sensor is a passive sensor and does not rely on any external equipment except ambient light, one of the most important features of visual navigation is the autonomy. With the improvement of computational capabilities, visual navigation can be applied to many important applications in various fields, for instance, robot navigation [1], unmanned aerial vehicles [2], and virtual or augmented reality.

Visual odometry (VO) was first raised by Nister et al. [3] and it has become a widely-used pose estimation method. Typical VO detects and extracts feature points from a series of images that were captured by camera, then matches feature points and calculates relative pose to estimate the relative ego-motion of camera. VO can be classified based on the number of cameras into monocular VO, stereo (binocular) VO [4], and multi-camera VO [5]. The main difference is that stereo and multi-camera VO can get absolute scale information in application while monocular VO dose not, and therefore requires a more complex initial process. Thus, the stereo VO is usually the preferable choice in practical navigation

Micro electro mechanical systems inertial measurement unit (MEMS-IMU) is also a common sensor in robots, unmanned aerial vehicles, and other moving carriers to estimate ego-motion [6,7]. It is mainly composed of accelerometers and gyroscopes, which are respectively used to obtain the acceleration and angular velocity of the carrier. Its high frequency provides precious motion information filling the interval gap of lower frequency associated vision sensors. Through using the two integrals of the acceleration and angular velocity, the attitude of the carrier can be measured. It also does not rely on any external information, can work in all conditions at any time, and has high data update rate, short-term accuracy and stability.

In recent years, visual and inertial information are usually combined to estimate the six degrees of freedom (6DOF) pose. When compared to VO, visual inertial odometry (VIO) [4,8,9,10] makes good use of the visual sensors and the inertial sensors, thereby acquiring more precise and robust 6DOF pose estimation. That also makes VIO play an essential role in autonomous navigation, especially in GPS-denied environment. Besides, more and more mobile robots are navigating through VIO, owing to the recent hardware improvements in mobile central processing units (CPUs) and graphics processing units (GPUs) (e.g., NVIDIA Jetson TX2 (NVIDIA corporation, Santa Clara, CA, USA)).

**Figure 1:**
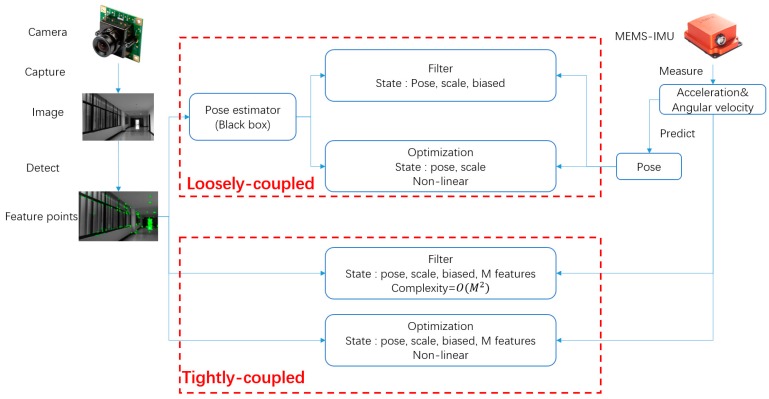
Loosely and tightly coupled visual inertial odometry (VIO).

The mainstream of existing VIO approaches can be classified into loose coupling and tight coupling [2,5,9,10,11] by type of information fusion shown in Figure 1. When the system is loosely-coupled, both inertial and visual information are seen as independent measurements. The process of visual pose estimation, regarded as a black box, is only used to update a filter to restrain the inertial measurement unit (IMU) covariance propagation. By contrast, tight coupling considers the interaction of all measurements of sensors information before pose estimation, thereby achieving higher accuracy than loose coupling.

Recently loosely-coupled stereo VIO systems are mostly based on Kalman filter and its derivatives. Tardif, et al. [12] proposed an EKF-based stereo VIO deployed on a moving vehicle. It used inertial information to predict the state and the stereo VO motion estimation as observations to get high frequency positioning information. Nevertheless, all of the states forecasted by inertial information, the covariance is sensitive to the IMU’s bias and drift. Liu, et al. [13] presented a stereo VIO that carried out the orientation and position estimation with three filters. It fused the accelerometer and gyroscope to estimate a drift-free pitch and roll angle then fused VO and IMU to estimate motion. Nevertheless, its filtering architecture was complex and not in real-time. Schmid, et al. [14] proposed a real-time stereo VIO. It computed high quality depth images and estimated the ego-motion by key-frame based VO and fused with the data of inertial information. However, it did not take the stereo VO’s failure into account. All loosely-coupled stereo VIO systems share the disadvantage that the stereo VO’s and IMU’s covariance were independent and cannot reflect the entire error.

Recently tightly-coupled stereo VIO systems mainly use a filtering-based [15] or optimization-based [16] approach. Filtering-based methods propagated the mean and covariance in kalman-filtering framework, together with feature points and IMU’s error. Sun, et al. [11] presented a filter-based stereo VIO system using the multi-state constraint kalman filter (MSCKF) [15] applied on an unmanned aerial vehicle. The system focused on lower computation costs. Ramezani, et al. [17] presented a stereo VIO system that was based on MSCKF and applied on vehicle, focusing on highly precise positioning. However, approaches above had high dimensional states vector and lack of robustness. The target of the optimization-based approach target was to minimize an energy function with a non-linear optimization by gauss-newton algorithm through frameworks, such as g2o [18] and ceres [19]. Usenko, et al. [4] presented a direct stereo VIO system estimated motion by minimizing a combined photometric and inertial energy function. It employed semi-dense depth maps instead of sparse feature points. Nevertheless, the inertial stability easily influenced by visual error and fault-tolerant method is simple consideration.

Subject to visual limitation, visual navigation is easily influenced when facing large scene changes that are caused by fast angular motion and low or dynamic light. To avoid positioning interruption, a fatal failure in robot navigation, current research mainly focuses on changing the feature descriptor to enhance the robustness of VO. Alismail, et al. [20] proposed new binary descriptors to achieve robust and efficient visual odometry with applications to poorly lit subterranean environments. However, the descriptors utilized information just from the images. When fast angular motion causes an image to be blurred or the environment is dark, the VO is doomed to fail. That will result in serious consequences.

To achieve satisfactory performance of VO withstanding all the limitations mentioned above, a fault-tolerant adaptive extended kalman filter (FTAEKF) framework integrated with a stereo-camera and a MEMS-IMU is proposed in this paper. The use of an EKF or one of its variants has been favored and extensively employed to fuse inertial and vision data, essentially to resolve pose estimation problem. When compared to traditional loose and tight VIO framework, both robustness and accuracy are under orders. Our main contributions are as follows:

- A stereo VIO with MEMS-IMU aided method is proposed in the framework. MEMS-IMU pre-integration constraint from prediction model is used to constrain a range of candidate feature points searching and matching. The constraint also set as to optimize the initial iterator pose to avoid local optimum instead of adding MEMS-IMU measurements error joint optimization.
- An adaptive method is introduced to adjust measurement covariance according to motion characteristic. Besides, a novel fault-tolerant mechanism is used to decide whether stereo VIO pose estimation is reliable by comparing it with MEMS-IMU measurements.

An improved stereo VIO method based on ORB-SLAM2 [21] (a visual-only stereo SLAM system demonstrated with its superior performance) is proposed in the framework. The framework can be easily integrated with any other stereo VO method. Because the computation process of MEMS-IMU pre-integration and initial iteration point prediction are mostly independent with the stereo VO.

The remainder of this paper is structured as follows: The definitions of coordinates and some symbols are presented in Section 2.1. The stereo VIO system aided by MEMS-IMU is introduced in Section 2.2. The FTAEKF is presented in Section 2.2.3. Experiment and evaluation of the proposed method are shown in Section 3, followed by discussion in Section 4.

## 2. Materials and Methods

### 2.1. Coordinates and Notations

**Figure 2:**
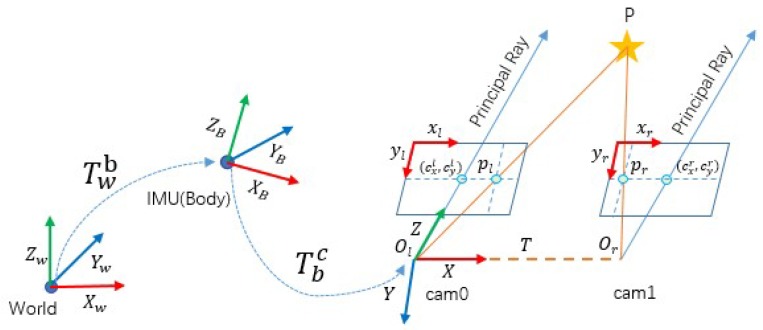
An illustration of coordinate system.

The four coordinates that were used in our framework are shown in Figure 2, The world frame W is defined as ENU (east-north-up) by axes $X_{W}$, $Y_{W}$, and $Z_{W}$, with $Z_{W}$ opposite to gravity, $Y_{W}$ points forward. The IMU frame, coincided with the body frame B also defined as ENU is attached to the center of MEMS-IMU with $Z_{B}$ pointing upward and $Y_{B}$ points forward. The camera frame C is set at the coordinate of left camera with $Z_{C}$ forward and $Y_{C}$ points downward. C is rigid relative pose with B. The relative pose is calibrated in advance.

The rotation matrix of framework is modeled by ZYX Euler angles. To get from *w* to *b*, rotates about $Z_{W}$, $Y_{W}$, and $X_{W}$ axes in turn, by the yaw angle ψ the pitch angle γ and the roll angle θ, respectively. The transformation matrix T is $T=\left[\begin{array}{cc} R & t\\ 0 & 1 \end{array}\right]$, where R∈SO(3) denotes the rotation matrix, and the rotation matrix $R_{w}^{c}$ represents from *w* to *c*. $t={\left(p_{x},p_{y},p_{z}\right)}^{T}$ denotes the translation vector. Vectors in the camera, body and world frames are defined as ${\left(\cdot \right)}^{c}$, ${\left(\cdot \right)}^{b}$ and ${\left(\cdot \right)}^{w}$, respectively. The transformation matrix from *w* to *b* is $T_{w}^{b}$, *b* to *c* is $T_{b}^{c}$.

### 2.2. Framework of Fault-Tolerant with Stereo-Camera and MEMS-IMU

**Figure 3:**
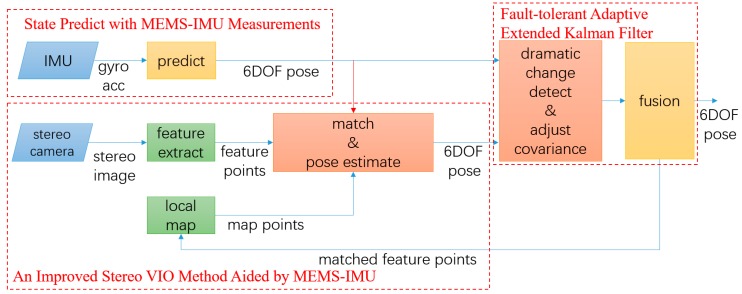
Framework of the proposed method. (the dark red blocks and red line are the difference between traditional VIO framework. the blue blocks represent the source from micro electro mechanical systems inertial measurement unit (MEMS-IMU) and stereo-camera. the green blocks represent traditional VO and dark yellow blocks represent MEMS-IMU measurements aided).

The pipeline of the proposed framework is illustrated in Figure 3. The aim of the proposed framework is to get robust and precise motion estimation in a hostile environment. The loop closing and full bundle adjustment in ORB-SLAM2 are not involved in this paper. Our contributions are mainly on the dark red block and the red arrow.

The stereo-camera and MEMS-IMU are tightly-coupled based on FTAEKF. The pre-integration of MEMS-IMU measurement confines the range of searching and matching feature points, and fault tolerance. Different from the traditional VIO method, the pre-integration of MEMS-IMU measurements is used to optimize the initial iterate point of pose estimation. It is also used to decide whether the result of pose estimation is credible to detect fault. Besides, to reflect the accumulated drift error, the observation covariance is adaptive according to motion characteristics. It combines the good properties of both loosely-coupled and tightly-coupled approaches. In this framework, the independence of stereo VO maximized. The framework has a good level of fault tolerance. It can function properly, even under stereo VIO failure, and then recover the whole system. This is because the framework allows a limited amount of independence and stereo VIO system avoids scale ambiguity in the monocular VO system. The details are described below.

#### 2.2.1. State Predict with MEMS-IMU Measurements

The framework of FTAEKF is based on an iterated EKF where the state prediction is driven by IMU measurements. The system states $x\in {\mathbb{R}}^{16\times 1}$ of VIO consists of number of states:

(1) $$x={\left(q^{w},p^{w},v^{w},\beta_{g}^{b},\beta_{a}^{b}\right)}^{T}\;$$

Namely, $q_{}^{w}={\left(q_{0}^{},q_{1}^{},q_{2}^{},q_{3}^{}\right)}^{T}$ is the attitude in quaternions, reflecting the world frame (W) to the body frame (B). $p_{}^{w}={\left(px_{}^{w},py_{}^{w},pz_{}^{w}\right)}^{T}$ is the position and $v_{}^{w}=\left(v_{{}_{x}}^{w},v_{{}_{y}}^{w},v_{{}_{z}}^{w}\right)$ is the velocity expressed in the world frame, $\beta_{g}^{b}$ and $\beta_{a}^{b}$ are the biases of three-axis gyroscopes and three-axis accelerometers, respectively. The measurements from gyroscope and accelerometer are denoted as $\eta_{wb}^{b}$ and $a_{wb}^{b}$, respectively.

The prediction model vector $\dot{x}={\left({\dot{q}}_{}^{w},{\dot{p}}_{}^{w},{\dot{v}}_{}^{w},{\dot{\beta }}_{g}^{b},{\dot{\beta }}_{a}^{b}\right)}^{T}$ is defined as:

(2) $$\begin{array}{l} {\dot{q}}^{w}=\frac{1}{2}\Omega \left({\hat{\eta }}_{wb}^{b}\right)q^{w}\\ {\dot{p}}^{w}=v^{w}\\ {\dot{v}}^{w}=C_{b}^{w}\left(a_{wb}^{b}-\beta_{a}^{b}\right)+g^{w}\\ {\dot{\beta }}_{g}^{b}=0\\ {\dot{\beta }}_{a}^{b}=0 \end{array}$$

with $C_{b}^{w}$ representing the rotation matrix from B to W, the instantaneous angular velocity of B relative to W expressed in coordinate frame B ${\hat{\eta }}_{wb}^{b}$ and the quaternion update matrix $\Omega \left({\hat{\eta }}_{wb}^{b}\right)$ are defined as: ${\hat{\eta }}_{wb}^{b}=\eta_{wb}^{b}-\beta_{g}^{b}$,

(3) $$\Omega \left({\hat{\eta }}_{wb}^{b}\right)=\left[\begin{array}{cccc} 0 & -{\hat{\eta }}_{wbx}^{b} & -{\hat{\eta }}_{wby}^{b} & -{\hat{\eta }}_{wbz}^{b}\\ {\hat{\eta }}_{wbx}^{b} & 0 & -{\hat{\eta }}_{wbz}^{b} & {\hat{\eta }}_{wby}^{b}\\ {\hat{\eta }}_{wby}^{b} & {\hat{\eta }}_{wbz}^{b} & 0 & -{\hat{\eta }}_{wbx}^{b}\\ {\hat{\eta }}_{wbz}^{b} & -{\hat{\eta }}_{wby}^{b} & {\hat{\eta }}_{wbx}^{b} & 0 \end{array}\right]\;$$

In proposed framework, the pre-integration of MEMS-IMU measurements is obtained through the prediction model.

#### 2.2.2. An Improved Stereo VIO Method Aided by MEMS-IMU

In this part, the pre-integration of MEMS-IMU measurements is used to aid the stereo VO system. The stereo VIO system that was employed in this paper is based on ORB-SLAM2 with good performance.

**Figure 4:**
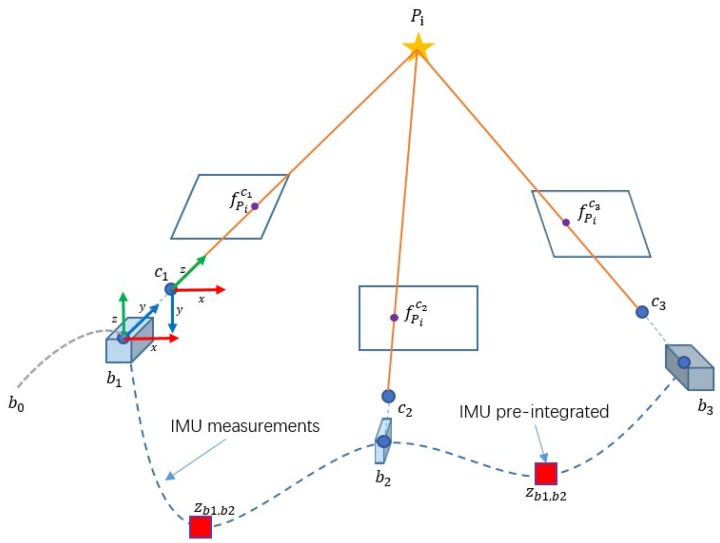
The process of improved stereo VIO method aided by MEMS-IMU. The inertial measurement unit (IMU) measurements are pre-integrated to predict position of feature points.

Both original feature based VO and VIO use brute-force or bag of words (BOW) matchers to match extracted feature points within reference frame and current frame These matchers take the descriptor of one feature in current frame and are matched to all other features in reference frame using hamming distance calculation. The closest one is returned. As a result, the pose estimation produced error when false matching occurred frequently in a hostile environment due to the close hamming distance of similar descriptor. In our approach, the MEMS-IMU measurements are pre-integrated to aid stereo VIO through constraining matching and predicting initial iteration pose. The process of this part shown in Figure 4.

Traditionally, the initial frame pose of stereo VO is configured as world frame. However, it hardly reflects physical truth. As shown in Figure 4, VIO initialized coordinate with MEMS-IMU forward as initial heading and aligns geographic coordinate system through gravity. The stereo VIO pose is compensated by $T_{w}^{b_{1}}$ from the MEMES-IMU measurement.

(4) $$T_{w}^{b_{1}}=\left[\begin{array}{cc} R_{w}^{b_{1}} & t_{w}^{b_{1}}\\ 0 & 1 \end{array}\right],R\in SO(3),t\in {\mathbb{R}}^{3\times 1}\;$$

where $R_{w}^{b_{1}}$ is the rotation matrix and $t_{w}^{b_{1}}$ are the translation matrix from *w* to $b_{1}$ when VIO obtains the first image. The time interval between the image and closest MEMS-IMU measurement can be ignored due to high frequency of MEMS-IMU and low dynamic condition in beginning.

When the first stereo image is retrieved from camera, ORB feature points are extracted and matched with left and right image to estimate the depth through epipolar and disparity constraints. Then initial three-dimensional (3D) feature points in C are generated and projected based on initial pose. When a new frame was obtained from the stereo-camera, the 3D feature points are reconstructed then matched to the reference frame 3D feature points with ORB descriptors. In order to avoid the false matching caused by similar descriptors in a hostile environment. We introduce MEMS-IMU pre-integration constraint, which confined the searching and matching region to get more correct matching.

**Figure 5:**
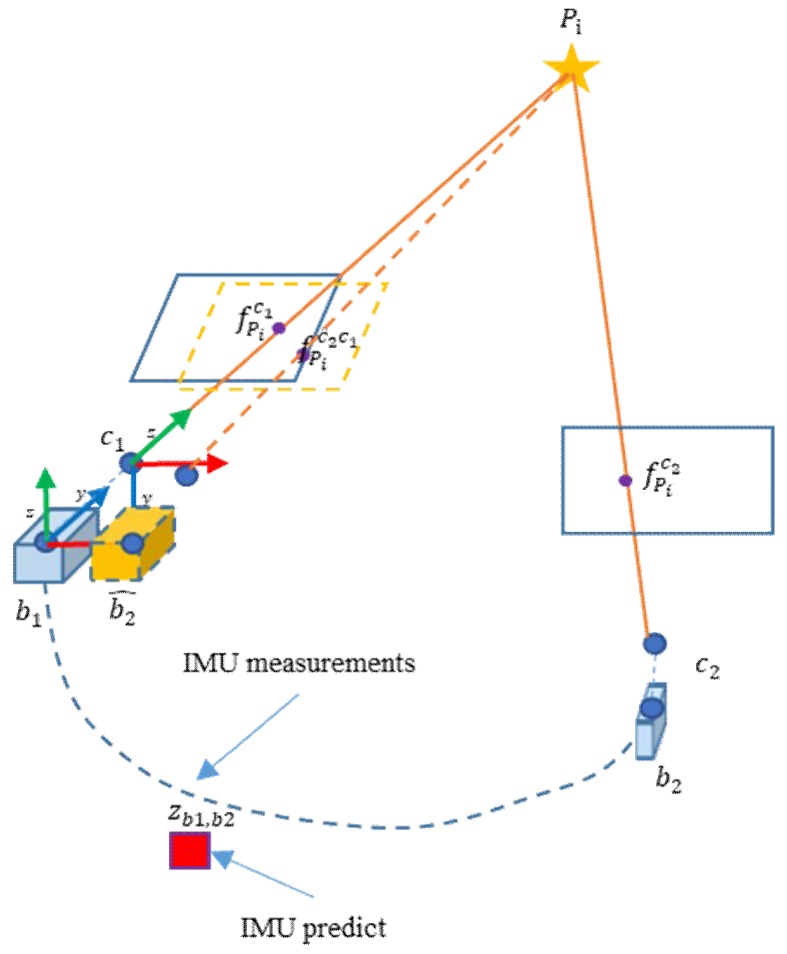
An illustration of predicting searching region with pre-integrating measurements of MEMS-IMU.

As shown in Figure 5, a point $P_{i}$ is observed by two consequent frames that obtain two feature points $f_{P_{i}}^{c_{1}}$, $f_{P_{i}}^{c_{2}}$. The feature point in current frame can be project to last frame with MEMS-IMU pre-integration. The coordinates in the pixel coordinates of both feature points $f_{P_{i}}^{c_{1}}$ and $f_{P_{i}}^{c_{2}c_{1}}$ are close after reprojection. We can match within bounds to decrease the workload and possibility of error.

In our approach, the MEMS-IMU pre-integration is obtained with the prediction model. MEMS-IMU measurements between two consequent frames at discrete time k−m, k predict MEMS-IMU pre-integration $\Delta \xi_{k-m,k}^{imu}={\left(\Delta q_{k-m,k}^{w},\Delta p_{k-m.k}^{w}\right)}^{T}$:

(5) $$\Delta \xi_{k-m,k}^{imu}={\sum_{i=k-m}^{k}\left[\begin{array}{ccc} \frac{1}{2}\Omega \left({\hat{\eta }}_{wb}^{b}\right)q_{i}^{w} & & \frac{1}{2}(v_{i-1}^{w}+v_{i}^{w}) \end{array}\right]}^{T}\Delta t\;$$

where $v_{i}^{w}$ denotes the velocity in *w* at time i, ${\hat{\eta }}_{wb}^{b}$ denotes the instantaneous angular velocity of B and $q_{i}^{w}$ denotes the quaternions from *w* to *b* at time i.

To reflect the motion of the camera, the pre-integration $\Delta \xi_{k-m,k}^{imu}$ needs to align with C:

(6) $$\begin{array}{l} T(\Delta \xi_{k-m,k}^{cam})=T_{b}^{c}T(\Delta \xi_{k-m,k}^{imu})T_{b}^{c}{}^{-1}\\ T(\Delta \xi_{k-m,k}^{imu})=\left[\begin{array}{cc} R_{k-m,k}^{b} & t_{k-m,k}^{b}\\ 0 & 1 \end{array}\right],T(\Delta \xi_{k-m,k}^{cam})=\left[\begin{array}{cc} R_{k-m,k}^{c} & t_{k-m,k}^{c}\\ 0 & 1 \end{array}\right] \end{array}$$

where $T(\Delta \xi_{k-m,k}^{cam})$ denotes the transformation matrix from time k−m to k in *c*, $T_{b}^{c}$ is the transformation matrix from *b* to *c*. $R_{k-m,k}^{b}$ is the quaternions $\Delta q_{k-m,k}^{w}$ expressed in rotation matrix, $t_{k-m,k}^{b}=C_{w}^{b}\Delta p_{k-m.k}^{w}$ is the translation vector in B, where $C_{w}^{b}$ is the rotation matrix from *w* to *b*.

After getting the coarse pose estimation of camera $\hat{T}(\Delta \xi_{k-m,k}^{cam})$, we can predict the camera pose by equation:

(7) $$\hat{T}\left(\xi_{k}^{cam}\right)=T(\Delta \xi_{k-m,k}^{cam})T\left(\xi_{k-m}^{cam}\right)=\left[\begin{array}{cc} \hat{R}\left(\xi_{k}^{cam}\right) & \hat{t}\left(\xi_{k}^{cam}\right)\\ 0 & 1 \end{array}\right]\;$$

For each 3D feature point of current frame, the matched feature points should near it. After predicting the coarse pose estimation, we project each feature point of current frame into the initial camera frame. The search for candidates only in a small range of each 3D feature points in local map. The range depends on the bias and noise of the MEMS-IMU. We do BOW matching between each feature point and its candidates to get matched feature point. Due to the confinement of the region, the error and the time consuming in searching and matching will reduce.

After getting the matched result, bundle adjustment optimization is performed to optimize the camera pose by minimizing the reprojection error between the matched 3D feature points $F^{i}\in {\mathbb{R}}^{3}$ in map and feature points $f^{i}\in {\mathbb{R}}^{3}$ in current frame. The i∈χ is a set of matched points:

(8) $$\left\{R,t\right\}=\underset{R,t}{\arg\min}\sum_{i\in \chi }\rho ({\left\|f_{\left(\cdot \right)}^{i}-\pi_{\left(\cdot \right)}(RF^{i}+t)\right\|}_{\sum }^{2})\;$$

where the ρ is the robust Huber cost function and ∑ is the covariance matrix associated to the scale of feature points, which is one when with stereo-camera. $\pi_{\left(\cdot \right)}$ is the projection functions monocular $\pi_{m}$, rectified stereo $\pi_{s}$ are defined, as follows:

(9) $$\pi_{m}\left(\left[\begin{array}{c} X\\ Y\\ Z \end{array}\right]\right)=\left(\begin{array}{c} f_{x}\frac{X}{Z}+c_{x}\\ f_{y}\frac{X}{Z}+c_{y} \end{array}\right),\pi_{s}\left(\left[\begin{array}{c} X\\ Y\\ Z \end{array}\right]\right)=\left(\begin{array}{c} f_{x}\frac{X}{Z}+c_{x}\\ f_{y}\frac{X}{Z}+c_{y}\\ f_{x}\frac{X-b}{Z}+c_{x} \end{array}\right)\;$$

where $\left(f_{x},f_{y}\right)$ is focal length, $\left(c_{x},c_{y}\right)$ is the principal point and b is the baseline, all is known in advanced.

**Figure 6:**
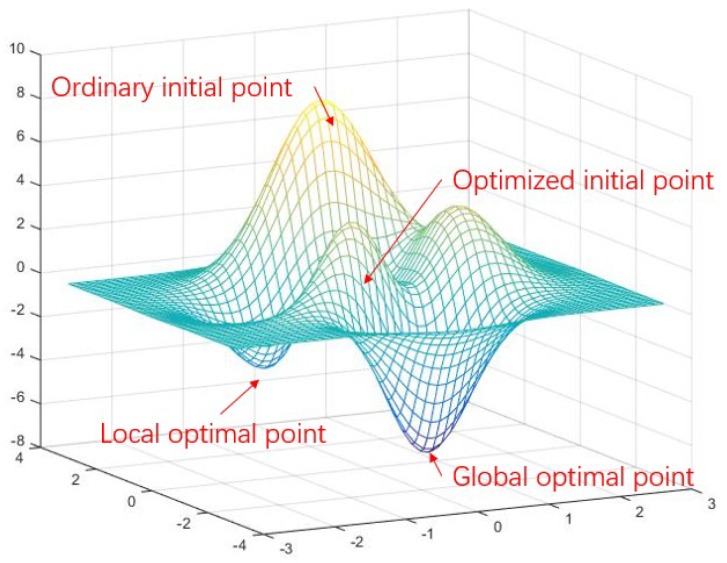
An illustration of association between initial point and result of optimization

However, the bundle adjustment to minimize the reprojection error is nonlinear. It cannot always get a global optimal point. As shown in Figure 6, VO falls into local optimum easily because the initial iteration point is last frame pose.

In our approach, the initial iteration pose is set as prediction of MEMS-IMU pre-integration $R=\hat{R}\left(\xi_{k}^{cam}\right)$ and $t=\hat{t}\left(\xi_{k}^{cam}\right)$ to get close to global optimal point. Then, stereo VIO 6DOF pose estimation is optimized in order to avoid local optimum.

#### 2.2.3. Fault-Tolerant Adaptive Extended Kalman Filtering

In this part, the FTAEKF is introduced to tolerant wrong stereo VIO pose estimation limited by the visual principle in a hostile environment.

1. Fault-tolerance with dramatic change detection

In some extreme cases, with fast motion in hostile environment, a large error of VIO pose estimation occurs because of the limited number in matched feature points or similar descriptor. The matcher matches feature points simply depending on the hamming distance. Therefore, a fault-tolerant method with MEMS-IMU measurements is introduced through dramatic change detection.

One way to detect the sudden step change, by comparing the number of matched points with threshold after eliminating exterior point in bundle adjustment, has been proposed before. However, this is an indirect technique. In some scenario, the number of matched points is large enough, but they mostly matched with wrong feature points and significant estimation error still occurs in this direction. Sudden step change detecting in VIO mostly consider setting a transformation threshold between two consequent frames. They all only detected faults without isolation lead to failure of the system. 

In this paper, a new approach using the detection function to detect and isolate dramatic change was proposed. As an accurate pose can be estimated from MEMS-IMU during a short period, the framework considered the MEMS-IMU pre-integration $\hat{T}(\Delta \xi_{k-m,k}^{cam})$ as a reference. It compares to final relative VIO pose estimation $T\left(\Delta \xi_{k-m,k}^{cam}\right)=T\left(\xi_{k}^{cam}\right)T{\left(\Delta \xi_{k-m}^{cam}\right)}^{-1}$ between time k and k−1 to detect dramatic change. If the value of detection function $f_{d}\geq 1$, then the dramatic change detection is deemed to occur. The detection function $f_{d}$ is defined as:

(10) $$\begin{array}{l} \Delta T\left(\Delta \xi_{k-m,k}^{cam}\right)=T\left(\Delta \xi_{k-m,k}^{cam}\right)\hat{T}{\left(\Delta \xi_{k-m,k}^{cam}\right)}^{-1}=\left[\begin{array}{cc} \Delta R_{k-m,k}^{cam} & \Delta t_{k-m,k}^{cam}\\ 0 & 1 \end{array}\right]\\ f_{d}=\sqrt{\frac{{\left(\Delta t_{k-m,k}^{cam}-t_{k-m,k}^{imu}\right)}^{T}\left(\Delta t_{k-m,k}^{cam}-t_{k-m,k}^{imu}\right)}{E_{\varepsilon t}{}^{2}}\cdot \frac{\varepsilon \psi_{k-m,k}{}^{2}+\varepsilon \theta_{k-m,k}{}^{2}+\varepsilon \gamma_{k-m,k}{}^{2}}{E_{\varepsilon \psi }{}^{2}+E_{\varepsilon \theta }{}^{2}+E_{\varepsilon \gamma }{}^{2}}} \end{array}$$

where the $\Delta T\left(\Delta \xi_{k-m,k}^{cam}\right)$ is the transformation difference estimation between pre-integration of MEMS-IMU measurements and VIO. $\varepsilon \psi_{k-m,k}$, $\varepsilon \theta_{k-m,k}$, and $\varepsilon \gamma_{k-m,k}$ are defined as: $\varepsilon \gamma_{k-m,k}=\Delta \gamma_{{}_{k-m,k}}^{imu}-\Delta \gamma_{{}_{k-m,k}}^{cam}$, $\varepsilon \theta_{k-m,k}=\Delta \theta_{{}_{k-m,k}}^{imu}-\Delta \theta_{{}_{k-m,k}}^{cam}$, and $\varepsilon \psi_{k-m,k}=\Delta \psi_{{}_{k-m,k}}^{imu}-\Delta \psi_{{}_{k-m,k}}^{cam}$. Where $\Delta \gamma_{{}_{k-m,k}}^{imu}$, $\Delta \theta_{{}_{k-m,k}}^{imu}$, and $\Delta \psi_{{}_{k-m,k}}^{imu}$ are the incremental relative attitude change estimated by MEMS-IMU measurements, $\Delta \gamma_{{}_{k-m,k}}^{cam}$, $\Delta \theta_{{}_{k-m,k}}^{cam}$, and $\Delta \psi_{{}_{k-m,k}}^{cam}$ are the incremental relative attitude change estimated by VIO.

The threshold $E_{\varepsilon t}$, $E_{\varepsilon \psi }$, $E_{\varepsilon \theta }$, and $E_{\varepsilon \gamma }$ are set up according to the drift of motion estimation by prediction using MEMS-IMU during one period of slam procedure, which is from discrete time k−m to k. As a more reliable pose can be estimated from MEMS-IMU during a short period of time, the transformation difference estimation between MEMS-IMU prediction and stereo VIO system estimation should be within this range.

In consideration of the drift of estimation by MEMS-IMU, the threshold $E_{\varepsilon t}$, $E_{\varepsilon \psi }$, $E_{\varepsilon \theta }$, and $E_{\varepsilon \gamma }$ change adaptively. As continuous change detected in hostile environment increases, $E_{\varepsilon t}$, $E_{\varepsilon \psi }$, $E_{\varepsilon \theta }$, and $E_{\varepsilon \gamma }$ are growing. $E_{\varepsilon t}$, $E_{\varepsilon \psi }$, $E_{\varepsilon \theta }$, and $E_{\varepsilon \gamma }$ are to be reinitialized with the original value if no environmental transition is detected.

2. Covariance adaptive filtering

Due to the change and accumulation of error in each process of pose estimation from VIO, the observation covariance from VIO is set to dynamic dependent upon the distance and motion characteristics to achieve better positioning accuracy. The observation covariance is adjusted to better represent practical situations.

VIO is a dead-reckon algorithm in which the error of stereo VIO pose estimation is accumulated by distance. A factor $\lambda_{d}$, related to the distance of stereo VIO $d_{}^{cam}$ reflect the error accumulating is introduced:

(11) $$\begin{array}{c} d_{}^{cam}=\sum_{i=1}^{k-1}\sqrt{t{\left(\Delta \xi_{i,i+1}^{cam}\right)}^{T}t\left(\Delta \xi_{i,i+1}^{cam}\right)}\\ \lambda_{d}=\sigma d_{}^{cam} \end{array}$$

where $t\left(\xi_{i,i+1}^{cam}\right)$ is the camera translation vector between time k and k+1 in C, σ is dependent on characteristics of the stereo VIO system.

Besides, the precision of stereo VIO pose estimation is also influenced obviously by motion characteristics. The field of view changes fast and the same feature points are reduced speedily when great angular change is made in a short time. MEMS-IMU measurements are more suitable and precise for the estimation and VIO is no longer reliable. Thus, a factor $\lambda_{a}$ is introduced to adapt the specialties of MEMS-IMU and stereo VIO.

(12) $$\lambda_{a}=\sum_{i=k-n}^{k}\sqrt{{\hat{\eta }}_{wb,i}^{b}{}^{T}{\hat{\eta }}_{wb,i}^{b}}\;$$

where ${\hat{\eta }}_{wb,i}^{b}$ is ${\hat{\eta }}_{wb}^{b}$ at time i, n is the size of the slide window.

When filtering, the error state vector used to correct the predicted state in filter is defined as follows:

(13) $$\delta X={\left(\delta q_{}^{w},\delta p_{}^{w},\delta v_{}^{w},\delta \beta_{g}^{b},\delta \beta_{a}^{b}\right)}^{T}\;$$

where, δX is the state vector composed by quaternions, position, velocity, and bias error.

With no dramatic change detecting in perceived environment, the predicted states are corrected by measurements information obtained from stereo VIO pose estimation. As no drift pitch or roll angle can be obtained through gravity correction, the observation model in proposed FTAEKF is as follows:

(14) $$\begin{array}{l} Z_{k}^{}=H_{k}^{}\delta X_{k}^{}+\mu_{k}\\ \mu_{k}={\left[\begin{array}{cccc} \lambda_{d}\varepsilon_{p_{x}}^{r} & \lambda_{d}\varepsilon_{p_{y}}^{r} & \lambda_{d}\varepsilon_{p_{z}}^{r} & \lambda_{a}\varepsilon_{\psi }^{r} \end{array}\right]}^{T}\\ Z_{k}={\left({\tilde{x}}_{{}_{k}}^{w}-{\bar{x}}_{{}_{k}}^{w},{\tilde{y}}_{{}_{k}}^{w}-{\bar{y}}_{{}_{k}}^{w},{\tilde{z}}_{{}_{k}}^{w}-{\bar{z}}_{{}_{k}}^{w},{\tilde{\psi }}_{{}_{k}}^{w}-{\bar{\psi }}_{{}_{k}}^{w}\right)}^{T}\\ {\bar{\psi }}_{k}^{w}=\tan^{-1}\left(\frac{2\left(q_{1,k}^{w}*q_{2,k}^{w}+q_{0,k}^{w}*q_{3,k}^{w}\right)}{1-2\left(q_{2,k}^{w}*q_{2,k}^{w}+q_{3,k}^{w}*q_{3,k}^{w}\right)}\right)\\ H_{k}^{}=\left[\begin{array}{cccccc} 0_{3\times 1} & 0_{3\times 1} & 0_{3\times 1} & 0_{3\times 1} & I_{3\times 3} & 0_{3\times 9}\\ \frac{\partial {\bar{\psi }}_{k}^{w}}{\partial q_{{}_{o,k}}^{w}} & \frac{\partial {\bar{\psi }}_{k}^{w}}{\partial q_{{}_{1,k}}^{w}} & \frac{\partial {\bar{\psi }}_{k}^{w}}{\partial q_{{}_{2,k}}^{w}} & \frac{\partial {\bar{\psi }}_{k}^{w}}{\partial q_{{}_{3,k}}^{w}} & 0_{1\times 3} & 0_{1\times 9} \end{array}\right] \end{array}$$

where $Z_{k}$ is the observation, ${\tilde{x}}_{{}_{k}}^{w}$, ${\tilde{y}}_{{}_{k}}^{w}$, ${\tilde{z}}_{{}_{k}}^{w}$, and ${\tilde{\psi }}_{{}_{k}}^{w}$ are the observation position and yaw in the world frame from the stereo VIO pose estimation, respectively, ${\bar{x}}_{{}_{k}}^{w}$,${\bar{y}}_{{}_{k}}^{w}$, ${\bar{z}}_{{}_{k}}^{w}$, and ${\bar{\psi }}_{{}_{k}}^{w}$ are the predicted position and yaw in the world frame from IMEMS-MU mechanization, respectively, $H_{k}^{}$ is the observation matrix and $\mu_{k}$ is the observation noise, which is adaptive.

When dramatic change occurred, MEMS-IMU measurements pre-integration will be used as pose estimation to isolate and tolerate fault. Since the pose estimated with MEMS-IMU during a short period of time is with sufficient accuracy, the stereo VIO system is reinitialized based on the MEMS-IMU pose in W at the closest time. The $\lambda_{a}$ and $\lambda_{d}$ is also reinitialized. That makes the framework with the ability to navigate even when stereo VIO system failed.

After filtering, the new matched feature points are projected to initial *c* to update the local map. The position of the same feature is represented using the average of position value.

When the dramatic change is detected, the local map points are cleared and the initial pose is set to MEMS-IMU pose in *w* with the closest time.

## 3. Results

### 3.1. Experiment Setup

#### 3.1.1. Equipment

**Figure 7:**
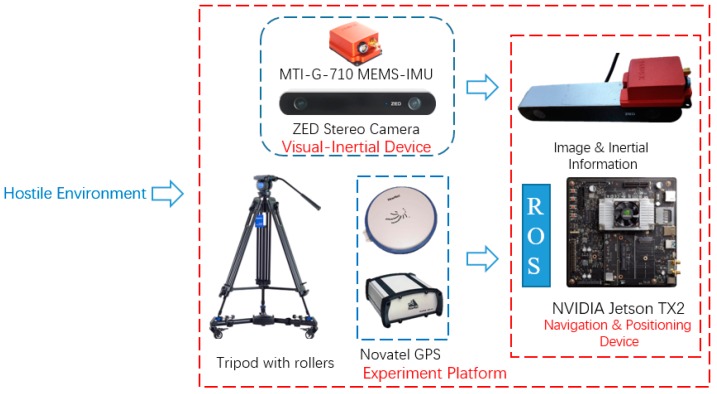
An illustration of platform. It consisted of ZED camera, MTI MEMS-IMU, Novatel GPS and Jetson TX2.

The equipment that we employed was based on commercial off the shelf shown in Figure 7. It consists of a ZED stereo camera, a Xsens MTI-G-710 MEMS-IMU, and a NVIDIA Jetson TX2. The ZED stereo camera resolution is set to 1280 × 720, baseline is 12cm and the frame rate at 15 HZ. The Xsens MTI-G-710 can measure the acceleration and angular velocity in body frame running at 200 HZ. The MEMS-IMU was mounted on left camera of ZED that was calibrated in advanced. The processing platform is NVIDIA Jetson TX2 with dual-core NVIDIA Denver2 and quad-core ARM Cortex-A57 running on Ubuntu 16.04. The Novatel OEM6 GPS receiver worked with GPS-RTK running at 1HZ as outdoor reference. All of the sensors were connected with TX2 through USB cable and the implementation is based on C++ with Robot Operating System (ROS) Kinetic. The sensors are mounted on a tripod with three rollers.

#### 3.1.2. Experiment Environment Description

**Figure 8:**
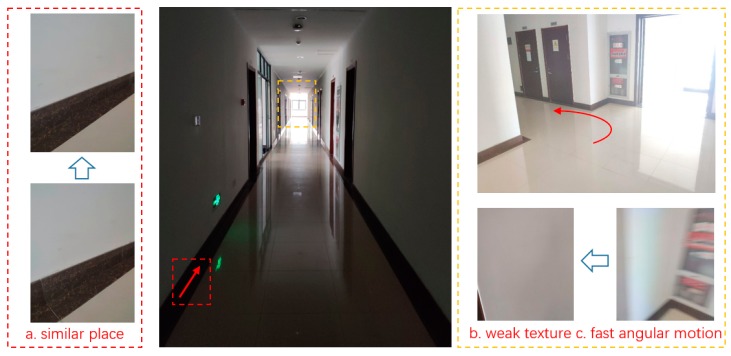
An illustration of the corridor where experiment carried on.

**Figure 9:**
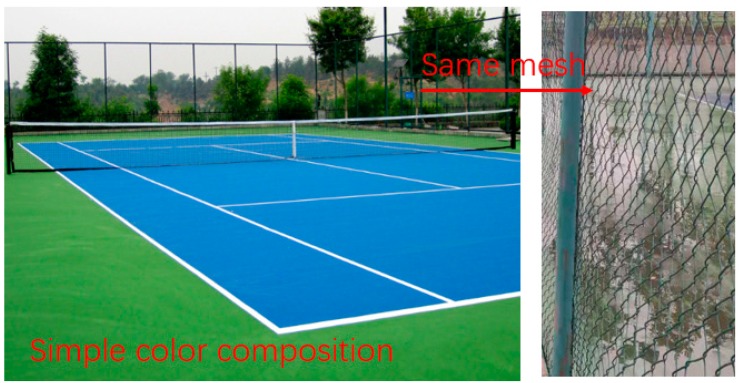
An illustration of the tennis court where experiment carried on.

In order to evaluate the performance of the proposed method under a hostile environment, the experiments were carried out in the corridor outside the laboratory and a tennis court in campus, as shown in Figure 8 and Figure 9. For the corridor, the wall of the corridor was sparse-feature. The make part of descriptors were similar. Ambient lighting in the corridor is unsatisfactory in some places, as it is bright near the window but is considerably darker elsewhere. The corridor plan is known in advance with the floor that consisted of fixed size tiles. Each tile is a square with sides of 60 cm. We pushed the tripod along the tile edge and obtained the ideal trajectory reference through a corridor plan. Some artificial mark points located at door and corner have been set in advance to evaluate the performance more comprehensively. It is regarded as the ideal path to evaluate the performance of the proposed framework. The yaw angle of MTI that was fused with magnetic is regarded as yaw angle reference. For the tennis court, the color of the ground was also simple and surrounded by similar meshes. The outdoor distance of feature was far beyond indoor environment. The reference of pose was obtained through GPS-RTK. Both environments can be considered as the hostile environment.

### 3.2. Experiments Results

We carried out a semi-physical simulation experiment to verify the performance of our proposed framework. The data was collected with the equipment and processed in platform. The proposed framework is compared against ORB-SLAM2, MSF-EKF [22], and VINS-Mono [23] in the experiments. The MSF-EKF based on the modular-sensor fusion framework by the University of Zurich is widely used to loosely couple inertial information and visual information. Moreover, the tightly-coupled VINS-Mono is high-performance and robust by the Hong Kong University of Science and Technology. Because the methods was multi-threaded and contained some random processing, the data took the 3σ bounds of results to eradicate any discrepancies.

#### 3.2.1. Experiment I: In Corridor

In experiment I, we pushed the tripod along the tile edge in the corridor. The experiment intended to assess the comprehensive performance of the proposed framework in an indoor hostile environment.

**Figure 10:**
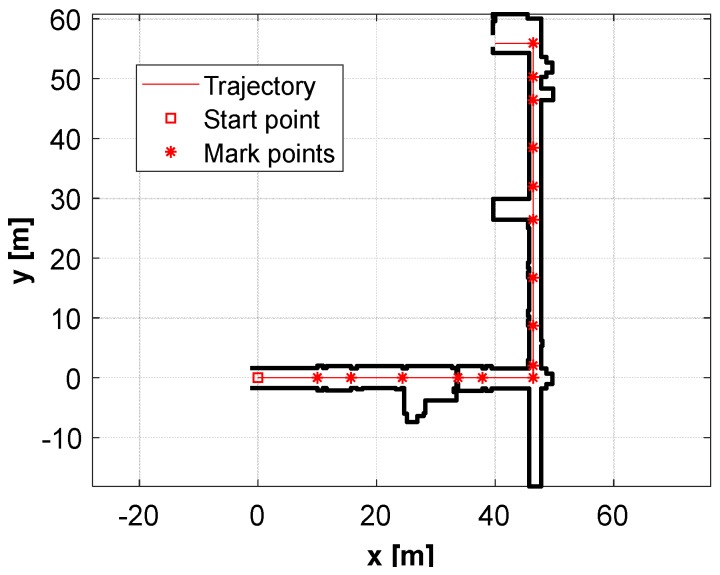
An illustration on the corridor plan, the ideal trajectory, and markers.

**Figure 11:**
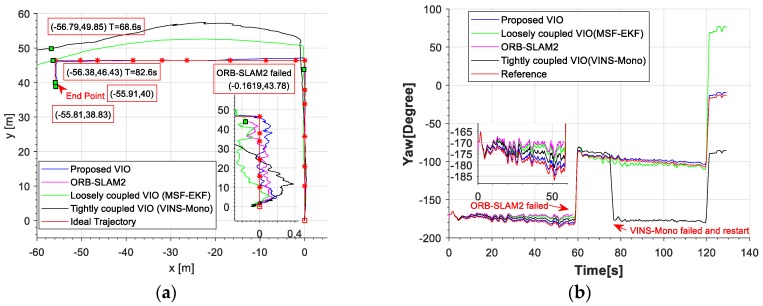
(**a**) An illustration of motion estimation results from different methods. (**b**) An illustration of Yaw angle estimated by different methods. ORB-SLAM2 failed due to few feature points and the noise of MEMS-IMU propagated speedily without measurements. The MEMS-IMU was meeting a corner causing fast angular velocity at 120 s. The noise of the gyroscopes propagated more speedily that causing sudden change in yaw angle difference with MSF-EKF.

**Figure 12:**
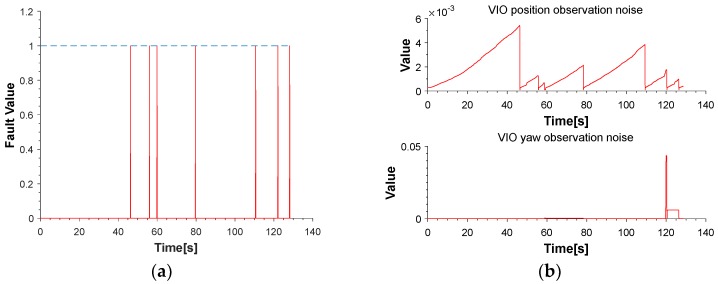
(**a**) The value of fault detect function demonstrates the dramatic change. (**b**) An illustration of the value of position and yaw observation noise.

**Figure 13:**
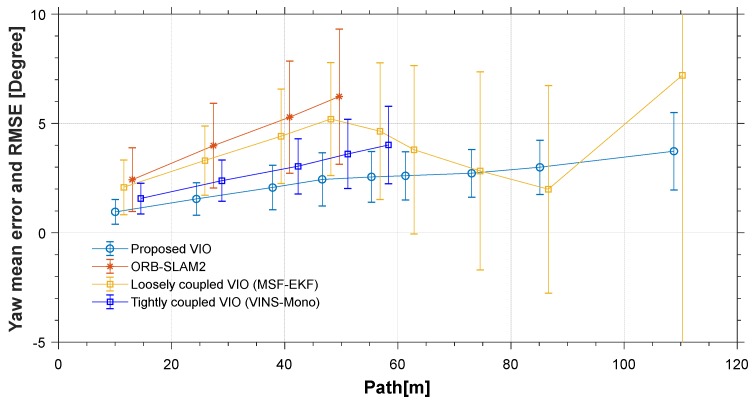
An illustration of value of yaw angle mean and RMSE from different methods. (VINS-Mono without output before initialization and value after system failing are ignored).

**Figure 14:**
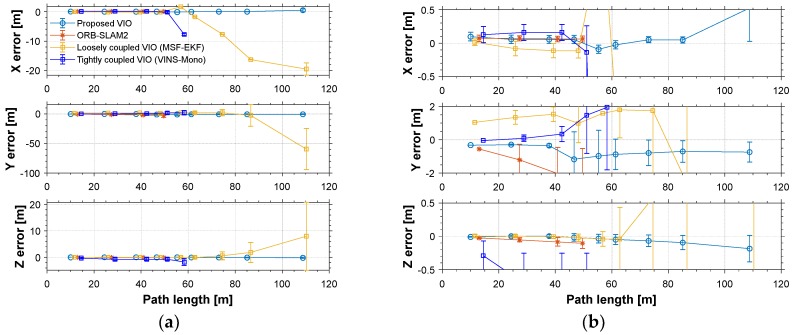
(**a**) The value of mean error and root mean square error (RMSE) of motion estimation from different methods. (**b**) An illustration of partial enlargement.

The red line is the ideal trajectory, as shown in Figure 10. The time at passing the mark points was recorded. The estimation of motion and yaw angle from different methods shown in Figure 11a,b. The position is projected onto X-Y plane. It was clear to see our proposed method achieved more accurate pose estimation. In addition, the value of fault illustrated seven dramatic changes that were detected by FTAKF in the experiment I in Figure 12a and the adaptive observation covariance is shown in Figure 12b. Moreover, the value of mean error and root mean square error (RMSE) of yaw angle and motion estimation from different methods, as shown in Figure 13 and Figure 14.

#### 3.2.2. Experiment II: In tennis court

In experiment II, we pushed the tripod along the edge of the tennis court. The experiment intended to evaluate the performance of the proposed framework in an outdoor hostile environment under the RTK position and heading reference.

**Figure 15:**
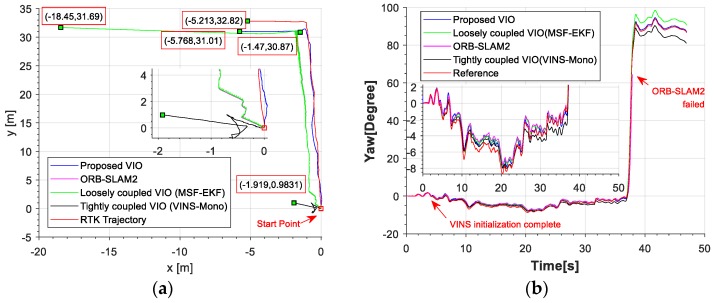
(**a**) An illustration of motion estimation results from different methods (**b**) An illustration of yaw angle estimated by different methods. (VINS-Mono without output before initialization).

**Figure 16:**
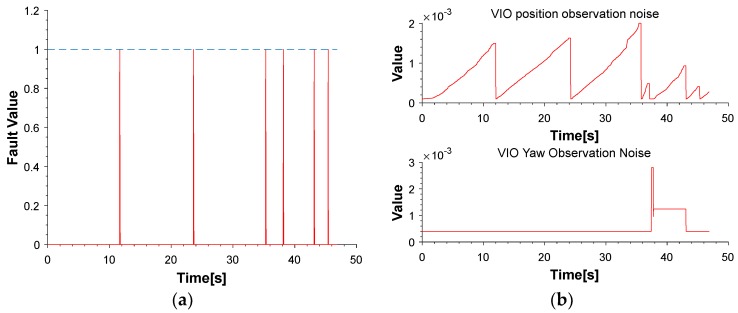
(**a**) The value of fault detect function demonstrates the dramatic change. (**b**) An illustration of the value of position and yaw observation noise.

**Figure 17:**
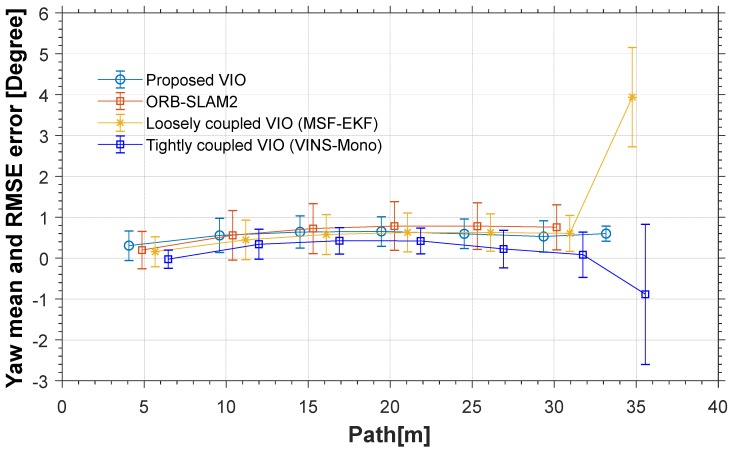
An illustration of value of yaw angle mean and RMSE from different methods. VINS-Mono without output before initialization.

**Figure 18:**
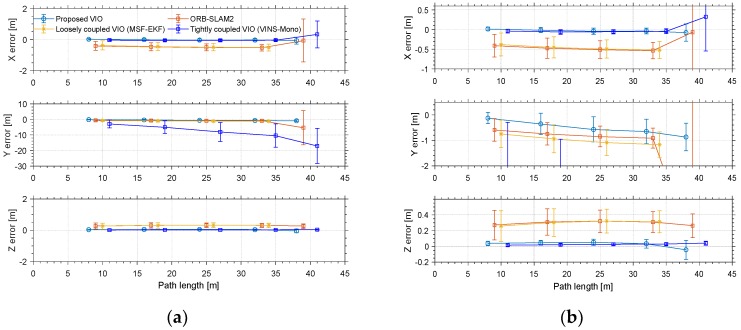
(**a**) The value of mean error and RMSE error of motion estimation from different methods. (**b**) An illustration of partial enlargement.

The red line is RTK trajectory as shown in Figure 15 with time synchronized through ROS. The estimation of motion and yaw angle from different methods shown in Figure 15a,b. Our proposed method achieved more accurate pose estimation. The value of fault illustrated six dramatic changes was detected by FTAKF in the experiment II in Figure 16a and the adaptive observation covariance is shown in Figure 16b. The value of mean error and RMSE of yaw angle and motion estimation from different methods shown in Figure 17 and Figure 18.

### 3.3. Experimental Analysis

#### 3.3.1. Accuracy Analysis

In the experiments, the accuracy of the proposed algorithm in the reconstructed trajectory is calculated as the RMSE with mark points and RTK references in Table 1 and Table 2. Moreover, the Euclidean distance between the last position of the estimated camera trajectory and the expected end point were calculated in Table 3 and Table 4. Value marked with an asterisk (*) was obtained before failure.

The accuracy for the experiments was depicted in above tables. The true length of different trajectories is, respectively, 108.8 m and 38 m, and the changes of reference yaw angle are 180° and 90°. As shown in Figure 11 and Figure 15, the stereo-camera and MEMS-IMU experienced different motions with smooth motion, fast rotational, and translational motion of indoor and outdoor. As both mean error and root mean square error of ORB-SLAM2, MSF-EKF, and VINS-Mono were larger than the proposed method in hostile environment. It is clearly seen that the estimated results from the proposed method in Experiment I and II were more accurate and robust than those from ORB-SLAM2, MSF-EKF, and VINS-Mono in Figure 13 and Figure 14 and Figure 17 and Figure 18. Pose estimation of both VO and VIO without fault tolerance were failed or divergent, which may cause fatal problems in robot navigation.

#### 3.3.2. Inertial Aided Matching and Fault Tolerance Analysis

Figure 11 and Figure 15 shows the pose estimation of two experiments from four different methods. ORB-SLAM2, MSF-EKF, and VINS-Mono produced large error in both position and yaw angle estimation under hostile environments. During experiments, systems including ORB-SLAM2 and VINS-Mono were in poor performance due to few feature or similar feature in hostile environment.

Moreover, ORB-SLAM2 failed because the number of feature points at corner lower than threshold. The failure of ORB-SLAM2 also caused divergence of MSF-EKF without VO output as measurement. With the number of feature points decreasing, the part of cost function occupied by each feature points was increasing. In addition, the influence of mismatch was increased, resulting in the divergence of a system. VINS-Mono failed by detecting much large translation between two frames in experiment I. For experiment II, the feature points in starting position of tennis court were too similar and far to produce enough disparity between two consequent frames. This situation caused the error in direction of x axis with ORB-SLAM2 and false initialization with VINS-Mono which tracking feature points through optical flow method.

**Figure 19:**
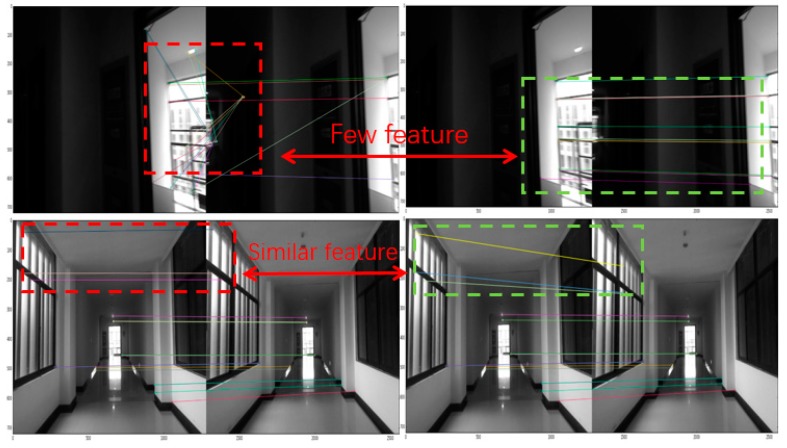
An illustration of wrong matching in hostile situation. Left image represented matching with all feature points in references frame and right confined matching by pre-integration.

The pre-integration of measurements of MEMS-IMU could constrain the region of matching to reduce incorrect candidate points that achieve better match result, as shown in Figure 19. Besides, the dramatic changes was detected shown in Figure 12a and Figure 16a, were isolated in the proposed framework that able to navigate properly in hostile environment. In addition, the adaptive noise of measurements shown in Figure 12b and Figure 16b make the proposed framework obtained more accurate pose estimation than traditional loosely-coupled VIO, such as MSF-EKF.

## 4. Conclusions

In this work, a novel fault-tolerant framework with stereo-camera and MEMS-IMU was proposed to obtain robust and precise positioning information in a hostile environment. MEMS-IMU measurements predict the camera motion and adaptive observation covariance noise are taken in the framework. It makes stereo VO motion estimation more precise when meeting hostile environment. A fault-tolerant mechanism is also introduced to detect and isolate the dramatic change in order to achieve more robust positioning information.

When comparing to traditionally loosely-coupled VIO systems that are not considered to detect the wrong measurements, our proposed method introduced an adaptive noise according to motion characteristics that obtain more precise positional information. For the tightly-coupled VIO systems, which introduced inertial error to obtain more robust and accurate positioning results, the relation between inertial error and visual error is not considered, which leads to the influence of inertial error estimation after the error of visual matching, resulting in the instability of the whole system. Our proposed framework isolated visual error, which was detected by comparing with more reliable inertial error, made the whole system more reliable and stable. The framework also maintains a certain degree of independence between framework and stereo VO system that can be easily integrated with other stereo VO system. By evaluating the results of experiments, the proposed VIO system has achieved a satisfactory performance in state estimation in a hostile environment.

In our future work, we hope to apply the inertial information to graph-pose optimization in order to realize the function of loop detection and optimization in hostile environment. We also hope to employ the method in more challenging environments.

## Figures and Tables

**Table 1:** RMSE (m) of motion estimation in different methods. (Value marked with an asterisk (*) was obtained before VO failure.)

Length (m)	Proposed Error	ORB-SLAM2 Error	MSF-EKF Error	VINS-Mono Error
Experiment I: 108.8	0.43(0.58 *)	0.94 *	16.57 (0.90 *)	1.80 *
Experiment II: 38	0.6(0.53 *)	0.75 *	3.94 (0.6 *)	0.88 (0.08 *)

**Table 2:** RMSE (°) of yaw angle estimation in different methods. (Value marked with an asterisk (*) was obtained before VO failure.)

Yaw Angle Change (°)	Proposed Error	ORB-SLAM2 Error	MSF-EKF Error	VINS-Mono Error
Experiment I: 180	4.52 (2.9 *)	3.13 *	21.84 (3.10 *)	3.0 *
Experiment II: 90	0.19 (0.38 *)	0.55 *	1.21 (0.44 *)	1.72 (0.56 *)

**Table 3:** Length accuracy (m).

Length (m)	Proposed Error	ORB-SLAM2 Error	MSF-EKF Error	VINS-Mono Error
Experiment I: 108.8	0.92, 0.8%	194.3, 179.9%	55.88, 51.4%	67.36, 67.4%
Experiment II: 38	1.89, 4.98%	4.22, 11.1%	13.3, 35.0%	32.0, 84.2%

**Table 4:** Yaw angle accuracy (°).

Yaw Angle Change (°)	Proposed Error	ORB-SLAM2 Error	MSF-EKF Error	VINS-Mono Error
Experiment I: 180	1.8, 1%	176.3, 97.9%	68.5, 38.1%	62.3, 34.6%
Experiment II: 90	0.37, 0.4%	22.17, 25%	3.98, 4.04%	5.95, 6.61%
